# Supplementary material for: Comprehensive Analysis of Co-Mutations Identifies Cooperating Mechanisms of Tumorigenesis
Source: Cancers (Basel). 2022 Jan 14;14(2):415. doi: 10.3390/cancers14020415 (PMC8774165; doi:10.3390/cancers14020415)
Supplement: Supplementary file 1 [file cancers-14-00415-s001.zip › SupplementaryFigures.pdf]

A

B

C

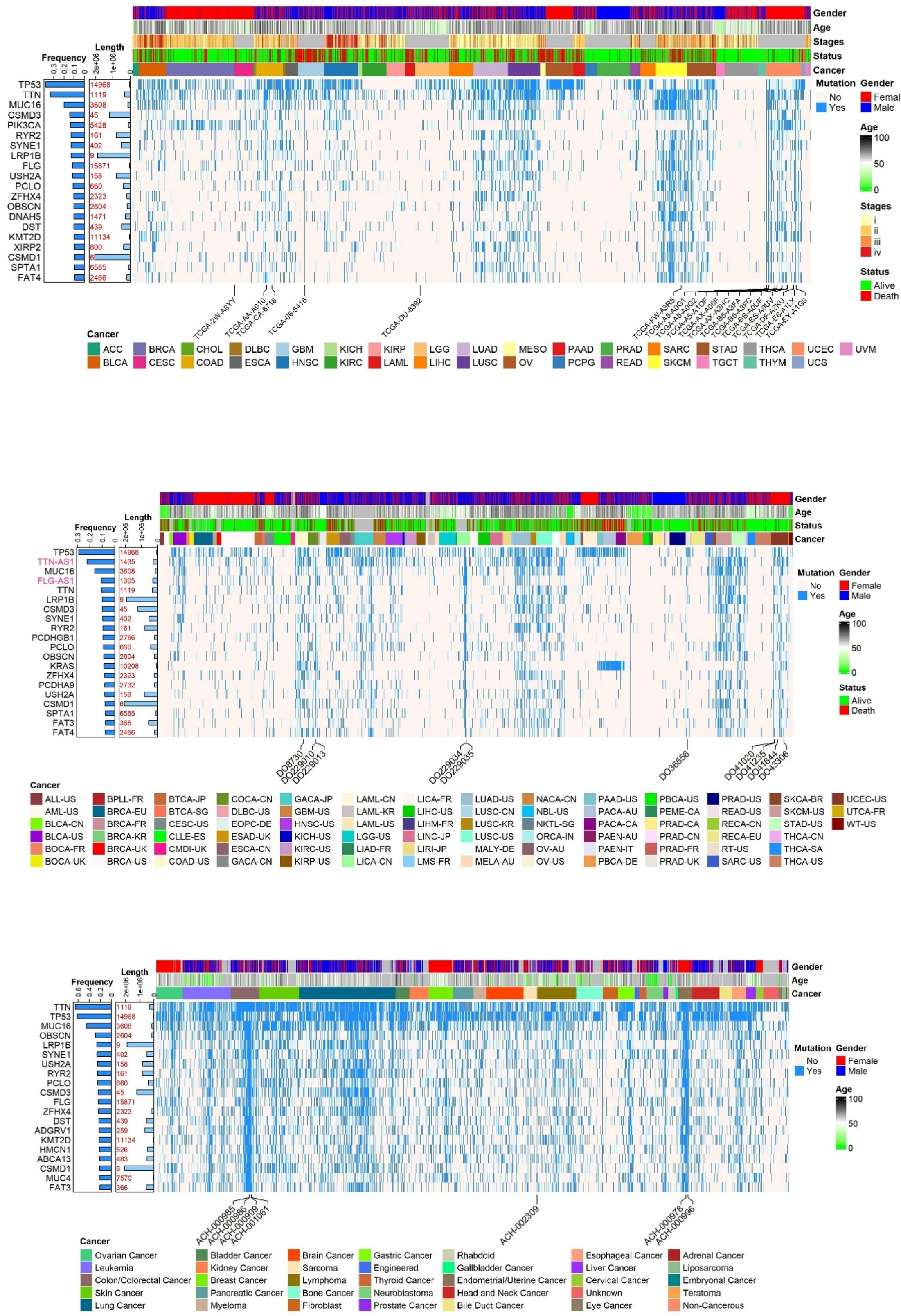

Gender
Age
Stages
Status
Cancer
Frequency
Length
Mutation
No
Yes
Female
Male
100
50
0
i
ii
iii
iv
Alive
Death
TP53
TTN
MUC16
CSMD3
PIK3CA
RYR2
SYNE1
LRP1B
FLG
USH2A
PCLO
ZFHX4
OBSCN
DNAH5
DST
KMT2D
XIRP2
CSMD1
SPTA1
FAT4
ACC
BLCA
BRCA
CESC
CHOL
COAD
DLBC
ESCA
GBM
HNSC
KICH
KIRC
KIRP
LAML
LGG
LIHC
LUAD
LUSC
MESO
OV
PAAD
PCPG
PRAD
READ
SARC
SKCM
STAD
TGCT
THCA
THYM
UCEC
UCS
UVM
TTN-AS1
FLG-AS1
PCDHGB1
KRAS
PCDHA9
FAT3
ALL-US
AML-US
BLCA-CN
BLCA-US
BOCA-FR
BOCA-UK
BPLL-FR
BRCA-EU
BRCA-FR
BRCA-KR
BRCA-UK
BRCA-US
BTCA-JP
BTCA-SG
CESC-US
CLLE-ES
CMDI-UK
COAD-US
COCA-CN
DLBC-US
EOPC-DE
ESAD-UK
ESCA-CN
GACA-CN
GACA-JP
GBM-US
HNSC-US
KICH-US
KIRC-US
KIRP-US
LAML-CN
LAML-KR
LAML-US
LGG-US
LIAD-FR
LICA-CN
LICA-FR
LIHC-US
LIHM-FR
LINC-JP
LIRI-JP
LMS-FR
LUAD-US
LUSC-CN
LUSC-KR
LUSC-US
MALY-DE
MELA-AU
NACA-CN
NBL-US
NKTL-SG
ORCA-IN
OV-AU
OV-US
PAAD-US
PACA-AU
PACA-CA
PAEN-AU
PAEN-IT
PBCA-DE
PBCA-US
PEME-CA
PRAD-CA
PRAD-CN
PRAD-FR
PRAD-UK
PRAD-US
READ-US
RECA-CN
RECA-EU
RT-US
SARC-US
SKCA-BR
SKCM-US
STAD-US
THCA-CN
THCA-SA
THCA-US
UCEC-US
UTCA-FR
WT-US
ADGRV1
HMCN1
ABCA13
MUC4
Ovarian Cancer
Leukemia
Colon/Colorectal Cancer
Skin Cancer
Lung Cancer
Bladder Cancer
Kidney Cancer
Breast Cancer
Pancreatic Cancer
Myeloma
Brain Cancer
Sarcoma
Thyroid Cancer
Bone Cancer
Fibroblast
Gastric Cancer
Engineered
Lymphoma
Neuroblastoma
Prostate Cancer
Rhabdoid
Gallbladder Cancer
Endometrial/Uterine Cancer
Head and Neck Cancer
Bile Duct Cancer
Esophageal Cancer
Liver Cancer
Cervical Cancer
Unknown
Eye Cancer
Adrenal Cancer
Liposarcoma
Embryonal Cancer
Teratoma
Non-Cancerous

Supplementary Figure S1. The twenty most frequently mutated genes in the three cancer data consortiums separately. A. The Cancer Genome Atlas (TCGA). B. International Cancer Genome Consortium (ICGC). C. Cancer Dependency Map (DepMap). Each row of the heatmap represents a gene, and each column represents a subject showing mutation in at least one gene. Gene names in black denote protein-coding genes, and gene names in pink denote non-coding genes. Two barplots are attached at the left side of the heatmap to annotate two distinct features of the mutated genes. One barplot denotes the mutation frequency of each gene across all cohorts of one same consortium; the other barplot visualizes the gene length with the corresponding rank specified. Available phenotypic variables of subjects were indicated with color bars on the top of the heatmap. Subjects bearing mutations in all 20 genes are identified below the heatmap.

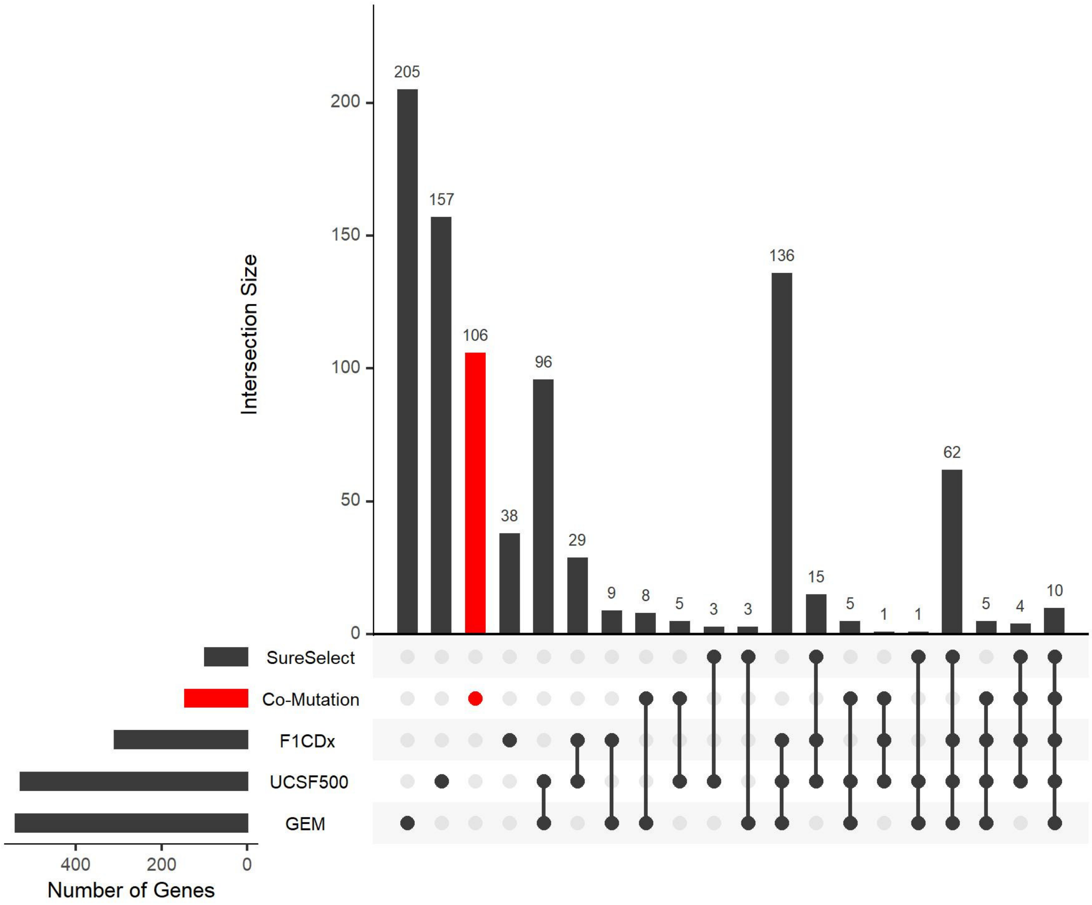

Supplementary Figure S2. Intersection analysis plot among five different cancer-related gene sets. The five gene sets included our identified co-mutation genes (red) and four clinical cancer gene panels (black), whose identification and gene number are depicted as horizontal barplot on the bottom left panel. The unitary, binary, tertiary, quaternary, and quinary intersection relations were illustrated with line segments on the bottom panel. The actual size of each intersection set formed by one, two, three, four, or five sets out of the total five was depicted in the vertical barplot on the top main panel. For example, the first verticle bar (205), represents the unique genes in GEM cancer gene panel and these 205 genes are not included in the other four gene sets. The last column

(10) represents the overall intersection of the five gene sets. The fourth to the last column (62), represents the intersection of the four cancer panels minus co-mutation gene sets. Thus, the four cancer gene panels share 62 + 10 = 72 genes. SureSelect, Agilent SureSelect. F1CDx, FoundationOne CDx. UCSF500, University of California San Francisco UCSF500. GEM, Ashion Genomic Enabled Medicine.
